# Account of Mr. Seguin's Remedy against Intermitting Fevers

**Published:** 1803-06-01

**Authors:** 


### *Account of* Mr. SEGUIN'S REMEDY *against* INTERMITTING FEVERS.

MR. SEGUIN, well known as a chemist, believes he has discovered a remedy in the animal *jelly*, against Intermitting Fevers. He was led to that discovery, as he affirms, in the following manner: His own children were afflicted with intermitting fever; he employed different sorts of bark without any effect, till using a certain sort of it, which cured the fever immediately. Being desirous to know the reason of that phenomenon, he submitted these different sorts of bark to a chemical analysis; by which means he discovered that the bark last employed, possesses, in a valuable degree, the power of precipitating *tanning*, a faculty not observed in the former. This fact led him to suppose, that the cause of intermitting fevers resides in the *tanning*, and that the bark cures that disease, in proportion as the disease contains *tanning*. In order to ascertain his opinion, he employed some other substances having the virtue of precipitating *tanning*, for the cure of intermitting fevers. He first tried *jelly*, and with such effect, that he saw no reason to proceed to farther experiments on other substances. He thus cured forty instances of intermitting fevers.

Having communicated his theory and his experiments to the National Institute of France, a committee was established to examine into the offered discovery. The Clinical School of Improvement (Ecole Clinique de perfectionement) at Paris, was chosen as the seat of experiments.—Some patients, affected with the above mentioned disease, were received there, and treated with jelly, and, with few exceptions, were all cured in a short time, either by the jelly or by the return of the spring season, but to which of these we will not decide. Whatever might be the result of these experiments, it still remains certain, that jelly is a very valuable *remedy* against hunger.

